# Supplementary material for: Impact of Antibiotic Prophylaxis on Surgical Site Infections in Cardiac Surgery
Source: Antibiotics (Basel). 2023 Jan 4;12(1):85. doi: 10.3390/antibiotics12010085 (PMC9854463; doi:10.3390/antibiotics12010085)
Supplement: Supplementary file 1 [file antibiotics-12-00085-s001.zip › antibiotics-2090933-supplementary.pdf]

antibiotics

Article

IMPACT OF ANTIBIOTIC PROPHYLAXIS ON SURGICAL SITE INFECTIONS IN CARDIAC SURGERY

Christian DE TYMOWSKI[1,2,3,4*], Tarek SAHNOUN[1], Sophie PROVENCHERE[1,8], Marylou PARA[5,6], Nicolas DERRE[1], Pierre MUTUON[7], Xavier DUVAL[6,8,11], Nathalie GRALL[9,11], Bernard Iung[6,10], Solen KERNEIS[6,11,12], Jean-Christophe LUCET[6,11,12], Philippe MONTRAVERS[1,6,13]

1. Department of Anaesthesiology and Surgical Intensive Care Unit, Groupe Hospitalier Bichat Claude Bernard, DMU PARABOL, Assistance Publique Hôpitaux de Paris, Paris, France
2. Université de Paris, Centre de Recherche sur l'Inflammation, INSERM UMR 1149, CNRS ERL8252, F-75018 Paris, France
3. Université Paris Cité, Laboratory of Excellence, Inflamex, F-75018 Paris, France
4. Department of Immunology, DHU Fire, Assistance Publique de Paris, Hôpital Bichat-Claude Bernard, Paris, France
5. Department of Cardiac Surgery, Groupe Hospitalier Bichat Claude Bernard, Assistance Publique Hôpitaux de Paris, Paris, France
6. Université Paris Cité, UFR Paris Nord, Paris, France
7. Service MSI, Groupe Hospitalier Bichat Claude Bernard, Assistance Publique Hôpitaux de Paris, Paris, France
8. INSERM Clinical Investigation Center 1425, Paris, France
9. Service de Bactériologie, Hôpital Bichat Claude Bernard, AP-HP, Université Paris Cité, 75018 Paris, France.
10. AP-HP, Cardiology Department, Bichat Hospital, Université Paris Cité, INSERM 1148, 46 rue Henri Huchard, 75018 Paris, France.
11. Université Paris Cité, Inserm, IAME, F-75018 Paris, France.
12. Infection Control Unit, Bichat-Claude Bernard Hospital, APHP, Paris, France
13. Physiopathologie et Epidémiologie des maladies respiratoires, INSERM UMR 1152, F-75018 Paris, France

* Correspondence: christian.detymowski@aphp.fr

Citation: Tymowski, C.d.; Sahnoun, T.; Provenchere, S.; Para, M.; Derre, N.; Mutuon, P.; Duval, X.; Grall, N.; Iung, B.; Kernéis, S.; et al. Impact of Antibiotic Prophylaxis on Surgical Site Infections in Cardiac Surgery. *Antibiotics* **2023**, *12*, 85. https://doi.org/10.3390/antibiotics12010085

Academic Editor: Jeffrey Lipman

Received: 26 November 2022
Revised: 27 December 2022
Accepted: 29 December 2022
Published: 4 January 2023

Copyright: © 2023 by the authors. Licensee MDPI, Basel, Switzerland. This article is an open access article distributed under the terms and conditions of the Creative Commons Attribution (CC BY) license (https://creativecommons.org/licenses/by/4.0/).

Supplementary Table S1: Perioperative characteristics of patients according to SSI subtype occurrence

	**WO SSI** N = 14,230 (96%)	**sSSI** N = 389 (2.6%)	**dSSI** N = 151 (1.0%)	*p value*
**Demography**				
Male	9,879 (69)	234 (60)	112 (74)	<0.001
Age (years)	66 [56-74]	68 [59-75]	70 [63-77]	<0.001
1st quartile < 56 years	3,595 (25)	72 (19)	25 (17)	<0.001
2nd quartile [56, 65] years	3,565 (25)	99 (25)	29 (19)	
3rd quartile [66-74] years	3,533 (25)	111 (29)	47 (31)	
4th quartile >74 years	3,537 (25)	107 (28)	50 (33)	
BMI (kg/m²)	26.1 [23.5-29.4]	29.1 [25.8-32.7]	27.8 [24.9-31.5]	<0.001
Obesity	3,051 (22)	169 (44)	55 (36)	<0.001
**Medical history**				
Smoking	5,102 (36)	130 (33)	67 (44)	0.056
Arterial hypertension	8,258 (58)	304 (78)	113 (75)	<0.001
Diabetes mellitus	3,721 (26)	249 (64)	66 (44)	<0.001
Insulin-dependent diabetes	1,096 (7.7)	125 (32)	24 (16)	<0.001
Non insulin-dependent diabetes	2,621 (18)	124 (32)	42 (28)	<0.001
Dyslipidaemia	7,084 (50)	259 (67)	89 (59)	<0.001
Chronic peripheral arterial insufficiency	1,659 (12)	103 (26)	39 (26)	<0.001
Neurovascular stroke	1,283 (9.0)	40 (10)	10 (6.6)	0.404
Cardiac insufficiency	631 (4.4)	22 (5.7)	17 (11)	<0.001
Ischaemic heart disease	6,953 (49)	331 (85)	104 (69)	<0.001
ESRD	173 (1.2)	13 (3.3)	5 (3.3)	<0.001
COPD	1,137 (8.0)	45 (12)	29 (19)	<0.001
Cirrhosis	144 (1.0)	2 (0.5)	2 (1.3)	0.535
**Preoperative data**				
B-blocker	8,238 (58)	289 (74)	93 (62)	<0.001
Angiotensin-converting enzyme inhibitors	6,865 (48)	235 (60)	84 (56)	<0.001
Statins	8,072 (57)	305 (78)	97 (64)	<0.001
Antiplatelet agent	8,008 (56)	314 (81)	108 (72)	<0.001
Aortic regurgitation	1,449 (10)	13 (3.3)	10 (6.6)	<0.001
Mitral regurgitation	1,771 (12)	13 (3.3)	10 (6.6)	<0.001
Aortic stenosis	3,589 (25)	42 (11)	33 (22)	<0.001
Mitral stenosis	1,022 (7.2)	10 (2.6)	6 (4.0)	<0.001
Surgical emergency	1,050 (7.4)	29 (7.5)	21 (14)	0.010
Acute infective endocarditis	751 (5.3)	4 (1.0)	4 (2.6)	<0.001
Prior cardiac surgery	1,314 (9.2)	18 (4.6)	13 (8.6)	0.008
Preoperative critical state	519 (3.6)	13 (3.3)	10 (6.6)	0.145
Preoperative MV	249 (1.7)	5 (1.3)	5 (3.3)	0.290
Preoperative catecholamine	301 (2.1)	6 (1.5)	5 (3.3)	0.382

Preoperative AKI	218 (1.5)	1 (0.3)	3 (2.0)	0.063
Preoperative haemoglobin (g/dl)	13.4 [12.1-14.5]	13.0 [11.8-14.2]	12.8 [11.7-14.2]	<0.001
Preoperative platelet count (G/l)	220 [182-266]	233 [190-283]	213 [180-274]	0.005
Preoperative prothombin ratio (%)	93 [82-100]	94 [84-100]	88 [77-100]	0.003
Preoperative creatinin (µg/l)	88 [75-107]	90 [74-116]	98 [84-120]	<0.001
Euroscore	2 [1-5]	2 [1-4]	3 [2-6]	<0.001
**Intraoperative data**				
VGA	1,720 (12)	33 (8.5)	21 (14)	0.075
ACC time	47 [36-66]	40 [33-52]	47 [36-63]	<0.001
CB time	60 [46-86]	49 [42-65]	59 [46-90]	<0.001
Isolated CABG	5,343 (38)	295 (76)	77 (51)	<0.001
Bimammary artery bypass	5,374 (38)	294 (76)	86 (57)	<0.001
CABG and valve surgery	1,319 (9.3)	34 (8.7)	27 (18)	<0.001
Isolated valvular surgery	5,721 (40)	43 (11)	30 (20)	<0.001
Thoracic aortic surgery	1,486 (10)	15 (3.9)	7 (4.6)	<0.001
Cardiac transplantation	202 (1.4)	3 (0.8)	9 (6.0)	<0.001
Post bypass catecholamine	5,301 (37)	169 (43)	69 (46)	0.005
Post bypass norepinephrine	1,790 (13)	47 (12)	12 (7.9)	0.223
Post bypass norepinephrine and dobutamine	1,168 (8.2)	48 (12)	13 (8.6)	0.014
**Postoperative data**				
MV duration (hours)	6 [5-10]	7 [5-12]	8 [6-26]	<0.001
Catecholamine duration (hours)	26 [10-56]	38 [14-74]	46 [18-84]	<0.001
Blood loss at 24 H (ml)	535 [380-740]	580 [428-790]	670 [480-1,000]	<0.001
Total blood loss (ml)	670 [480-945]	760 [570-980]	855 [571-1,392]	<0.001
Postoperative reintervention	363 (2.6)	389 (100)	151 (100)	<0.001
Surgical site infection	0 (0)	389 (100)	151 (100)	<0.001
Superficial SSI	0 (0)	389 (100)	0 (0)	<0.001
Deep SSI	0 (0)	0 (0)	151 (100)	<0.001
ICU LOS (days)	3 [2-5]	4 [2-8]	7 [3-23]	<0.001
Total LOS (days)	11 [8-16]	30 [10-43]	38 [23-58]	<0.001
D28 mortality	573 (4.0)	6 (1.5)	10 (6.6)	0.012
D90 mortality	750 (5.3)	25 (6.4)	27 (18)	<0.001

Continuous variables are expressed as the median and interquartile range (IQR) and were compared using the Mann–Whitney U test. Categorical variables are expressed as n (%) and were compared with Fisher's exact test.

BMI, body mass index; CABG, coronary artery bypass graft; CB, cardiopulmonary bypass; COPD, chronic obstructive pulmonary disease; ESRD, end-stage renal disease; ICU, intensive care unit; LOS, length of stay; MV, mechanical ventilation; SSI, surgical site infection; dSSI, deep SSI; sSSI, superficial SSI; VGA, vancomycin/gentamicin antibiotic prophylaxis.

Supplementary Table S2: Multivariate analysis of risk factors for superficial and deep SSI

**Predictors**	**Superficial SSI**			**Deep SSI**		
	**Estimate**	**CI**	**p**	**Estimates**	**CI**	**p**
Male	0.40	0.31 – 0.50	**<0.001**	1.01	0.68 – 1.51	0.942
Age (years)						
1st quartile < 56 y	ref	ref	-	ref	ref	-
2nd quartile [56, 65] y	0.76	0.54 – 1.06	0.102	0.92	0.52 – 1.63	0.769
3rd quartile [66-74] y	0.85	0.61 – 1.17	0.310	1.61	0.95 – 2.72	0.077
4th quartile >74 y	1.02	0.73 – 1.41	0.922	2.12	1.25 – 3.61	**0.005**
Obesity	2.27	1.82 – 2.83	**<0.001**	1.96	1.38 – 2.79	**<0.001**
Insulin-dependent diabetes	4.27	3.23 – 5.64	**<0.001**	1.59	0.97 – 2.58	0.064
Noninsulin-dependent diabetes	2.24	1.72 – 2.93	**<0.001**	1.26	0.84 – 1.89	0.268
Dyslipidaemia	0.81	0.64 – 1.04	0.094	0.87	0.61 – 1.26	0.463
Chronic peripheral arterial insufficiency	1.63	1.26 – 2.10	**<0.001**	1.69	1.13 – 2.51	**0.010**
Cardiac insufficiency	0.93	0.56 – 1.55	0.781	1.67	0.87 – 3.19	0.125
ESRD	2.00	1.06 – 3.78	**0.033**	1.73	0.61 – 4.89	0.298
COPB	1.24	0.88 – 1.75	0.223	2.22	1.45 – 3.39	**<0.001**
Acute infective endocarditis	0.62	0.22 – 1.76	0.368	0.52	0.15 – 1.79	0.304
Prior cardiac surgery	1.55	0.89 – 2.71	0.121	1.40	0.72 – 2.73	0.324
CPB time (/10 min)	0.96	0.92 – 1.00	0.052	1.06	1.01 – 1.10	**0.009**
CABG	2.86	1.79 – 4.58	**<0.001**	2.14	1.17 – 3.91	**0.013**
Bimammary artery bypass	2.24	1.50 – 3.34	**<0.001**	1.34	0.78 – 2.31	0.293
Cardiac transplantation	1.03	0.23 – 4.60	0.964	8.48	3.22 – 22.31	**<0.001**
Post-bypass norepinephrine and dobutamine	2.56	1.79 – 3.67	**<0.001**	0.54	0.27 – 1.10	0.089
**VGA**	**0.84**	**0.57 – 1.24**	**0.386**	1.22	0.73 – 2.02	0.444

CABG, coronary artery bypass graft; CB, cardiopulmonary bypass; COPD, chronic obstructive pulmonary disease; ESRD, end-stage renal disease; SSI, surgical site infection, VGA, vancomycin gentamicin antibiotic prophylaxis.

Supplementary Table S3: Multivariate analysis of risk factors for superficial and deep SSI in the propensity score analysis

**Predictors**	**Superficial SSI**			**Deep SSI**		
	**Estimate**	**CI**	**p**	**Estimates**	**CI**	**p**
Male	0.51	0.40 – 0.65	**<0.001**	2.00	1.24 – 3.24	**0.004**
Age (years)						
1st quartile < 56 y	ref	ref	-	ref	ref	-
2nd quartile [56, 65] y	0.94	0.67 – 1.32	0.727	0.47	0.28 – 0.78	**0.004**
3rd quartile [66-74] y	1.15	0.82 – 1.61	0.418	0.68	0.41 – 1.12	0.132
4th quartile >74 y	1.03	0.72 – 1.49	0.860	0.33	0.16 – 0.69	**0.003**
Obesity	2.76	2.18 – 3.49	**<0.001**	5.90	4.05 – 8.58	**<0.001**
Insulin-dependent diabetes	3.24	2.38 – 4.42	**<0.001**	1.46	0.73 – 2.92	0.287
Noninsulin-dependent diabetes	1.90	1.43 – 2.52	**<0.001**	0.98	0.56 – 1.71	0.949
Dyslipidaemia	0.57	0.44 – 0.74	**<0.001**	0.18	0.10 – 0.31	**<0.001**
Chronic peripheral arterial insufficiency	1.33	0.98 – 1.81	0.064	1.38	0.73 – 2.63	0.321
Cardiac insufficiency	0.98	0.58 – 1.66	0.939	3.75	1.89 – 7.45	**<0.001**
ESRD	4.73	3.18 – 7.03	**<0.001**	4.27	2.27 – 8.02	**<0.001**
COPB	1.91	1.41 – 2.57	**<0.001**	0.59	0.28 – 1.22	0.152
Acute infective endocarditis	0.98	0.67 – 1.43	0.921	4.05	2.52 – 6.49	**<0.001**
Prior cardiac surgery	1.04	0.67 – 1.61	0.850	2.14	1.34 – 3.41	**0.001**
CB time (/10 min)	0.96	0.93 – 0.99	**0.004**	0.99	0.95 – 1.03	0.691
CABG	3.48	2.27 – 5.32	**<0.001**	0.68	0.19 – 2.43	0.556
Bimammary artery bypass	1.55	1.04 – 2.31	**0.030**	4.29	1.14 – 16.18	**0.031**
Cardiac transplantation	0.80	0.34 – 1.89	0.608	2.49	1.05 – 5.90	**0.039**
Post-bypass norepinephrine and dobutamine	2.74	2.06 – 3.63	**<0.001**	0.40	0.23 – 0.69	**0.001**
**VGA**	0.76	0.52 – 1.11	0.150	1.28	0.77 – 2.15	0.339

CABG, coronary artery bypass graft; CB, cardiopulmonary bypass; COPD, chronic obstructive pulmonary disease; ESRD, end-stage renal disease; SSI, surgical site infection; VGA, vancomycin gentamicin antibiotic prophylaxis.

Supplementary Table S4: Perioperative characteristics of patients according to the type of prophylaxis in the sensibility analysis excluding patients with active endocarditis

	**Overall** N = 14,0 11 (100%)	**CA** N = 12,711 (91%)	**VGA** N = 1,300 (9%)	***p value***
**Demography**				
Male	9,662 (69)	8,940 (70)	722 (56)	<0.001
Age (years)	66 [57-74]	66 [57-74]	66 [56-75]	0.833
1st quartile < 56 years	3,391 (24)	3,053 (24)	338 (26)	0.246
2nd quartile [56, 65] years	3,499 (25)	3,190 (25)	309 (24)	
3rd quartile [66-74] years	3,544 (25)	3,233 (25)	311 (24)	
4th quartile >74 years	3,577 (26)	3,235 (25)	342 (26)	
BMI (kg/m²)	26.3 [23.7-29.4]	26.3 [23.7-29.4]	26.1 [23.3-29.6]	0.195
Obesity	3,153 (23)	2,847 (23)	306 (24)	0.377
**Medical history**				
Smoking	5,049 (36)	4,577 (36)	472 (36)	0.830
Arterial hypertension	8,349 (60)	7,608 (60)	741 (57)	0.046
Diabetes mellitus	3,909 (28)	3,547 (28)	362 (28)	0.964
Insulin-dependent diabetes	1,194 (8.5)	1,067 (8.4)	127 (9.8)	0.091
Noninsulin-dependent diabetes	2,711 (19)	2,477 (19)	234 (18)	0.196
Dyslipidaemia	7,240 (52)	6,638 (52)	602 (46)	<0.001
Chronic peripheral arterial insufficiency	1,730 (12)	1,572 (12)	158 (12)	0.824
Stroke	1,136 (8.1)	996 (7.8)	140 (11)	<0.001
Cardiac insufficiency	654 (4.7)	549 (4.3)	105 (8.1)	<0.001
Ischaemic heart disease	7,313 (52)	6,798 (53)	515 (40)	<0.001
ESRD	156 (1.1)	115 (0.9)	41 (3.2)	<0.001
COPD	1,145 (8.2)	985 (7.7)	160 (12)	<0.001
Cirrhosis	113 (0.8)	91 (0.7)	22 (1.7)	<0.001
**Preoperative data**				
B-blocker	8,465 (60)	7,790 (61)	675 (52)	<0.001
ACE inhibitor	7,018 (50)	6,398 (50)	620 (48)	0.070
Statins	8,312 (59)	7,650 (60)	662 (51)	<0.001
Antiplatelet agent	8,266 (59)	7,636 (60)	630 (48)	<0.001
Aortic regurgitation	1,114 (8.0)	968 (7.6)	146 (11)	<0.001
Mitral regurgitation	1,577 (11)	1,342 (11)	235 (18)	<0.001
Aortic Stenosis	3,622 (26)	3,244 (26)	378 (29)	0.005
Mitral stenosis	1,012 (7.2)	887 (7.0)	125 (9.6)	<0.001
Surgical emergency	930 (6.6)	802 (6.3)	128 (9.8)	<0.001
Acute infective endocarditis	0 (0)	0 (0)	0 (0)	
Prior cardiac surgery	1,126 (8.0)	927 (7.3)	199 (15)	<0.001
Preoperative critical state	371 (2.6)	277 (2.2)	94 (7.2)	<0.001
MV	145 (1.0)	91 (0.7)	54 (4.2)	<0.001
Catecholamine	209 (1.5)	151 (1.2)	58 (4.5)	<0.001

AKI	125 (0.9)	79 (0.6)	46 (3.5)	<0.001
Haemoglobin (g/dl)	13.5 [12.3-14.6]	13.6 [12.4-14.6]	12.8 [11.4-14.0]	<0.001
Platelet count (G/l)	220 [183-265]	219 [182-264]	226 [186-276]	0.002
Prothombin ratio (%)	94 [83-100]	94 [84-100]	92 [80-100]	<0.001
Creatinin (µg/L)	88 [75-106]	88 [75-106]	89 [75-114]	0.004
EuroSCORE II	2 [1-4]	2 [1-4]	3 [2-7]	<0.001
**Intraoperative data**				
ACC time	46 [36-64]	45 [36-64]	50 [37-71]	<0.001
CBP time	58 [46-83]	58 [45-82]	66 [48-97]	<0.001
Isolated CABG	5,714 (41)	5,387 (42)	327 (25)	<0.001
Bimammary artery bypass	5,740 (41)	5,387 (42)	353 (27)	<0.001
CABG and valve surgery	1,323 (9.4)	1,175 (9.2)	148 (11)	0.012
Isolated valvular surgery	5,176 (37)	4,564 (36)	612 (47)	<0.001
Thoracic aortic surgery	1,430 (10)	1,306 (10)	124 (9.5)	0.404
Cardiac transplantation	211 (1.5)	155 (1.2)	56 (4.3)	<0.001
Post-bypass catecholamine	5,073 (36)	4,504 (35)	569 (44)	<0.001
Post-bypass norepinephrine	1,724 (12)	1,625 (13)	99 (7.6)	<0.001
Post-bypass norepinephrine and dobutamine	1,068 (7.6)	927 (7.3)	141 (11)	<0.001
**Postoperative data**				
MV duration (hours)	6 [5-9]	6 [5-9]	7 [5-12]	<0.001
Catecholamine duration (hours)	25 [10-54]	24 [10-51]	34 [16-72]	<0.001
Blood loss at 24 H (ml)	540 [390-750]	545 [390-750]	482 [340-700]	<0.001
Total blood loss (ml)	680 [480-950]	680 [490-950]	630 [430-890]	<0.001
Reintervention	865 (6.2)	782 (6.2)	83 (6.4)	0.740
Surgical site infection	532 (3.8)	480 (3.8)	52 (4.0)	0.688
Superficial SSI	385 (2.7)	353 (2.8)	32 (2.5)	0.507
Deep SSI	147 (1.0)	127 (1.0)	20 (1.5)	0.069
ICU LOS (days)	3 [2-5]	3 [2-5]	4 [2-6]	<0.001
Total LOS (days)	11 [8-16]	10 [8-15]	13 [8-20]	<0.001
D28 mortality	477 (3.4)	410 (3.2)	67 (5.2)	<0.001
D90 mortality	655 (4.7)	559 (4.4)	96 (7.4)	<0.001

Supplementary Table S5: Multivariate analysis of risk factors for superficial and deep SSI in the sensitivity analysis excluding active endocarditis

Predictors	Superficial SSI			Deep SSI		
	Estimates	CI	p	Estimates	CI	p
Male	0.41	0.32 – 0.52	**<0.001**	1.08	0.71 – 1.62	0.728
Age (years)						
1st quartile < 56 y	ref	ref	-	ref	ref	-
2nd quartile [56, 65] y	0.75	0.54 – 1.05	0.089	0.99	0.55 – 1.78	0.975
3rd quartile [66-74] y	0.84	0.61 – 1.17	0.303	1.68	0.98 – 2.90	0.061
4th quartile >74 y	1.03	0.74 – 1.43	0.871	2.27	1.32 – 3.92	**0.003**
Obesity	2.23	1.78 – 2.79	**<0.001**	1.88	1.32 – 2.69	**0.001**
Insulin-dependent diabetes	4.33	3.27 – 5.73	**<0.001**	1.65	1.01 – 2.69	**0.045**
Noninsulin-dependent diabetes	2.22	1.70 – 2.90	**<0.001**	1.29	0.86 – 1.93	0.226
Dyslipidaemia	0.82	0.64 – 1.05	0.113	0.90	0.62 – 1.30	0.569
Chronic peripheral arterial insufficiency	1.64	1.27 – 2.12	**<0.001**	1.71	1.14 – 2.54	**0.009**
Cardiac insufficiency	0.95	0.57 – 1.58	0.838	1.47	0.75 – 2.88	0.267
ESRD	1.89	0.98 – 3.66	0.058	1.30	0.40 – 4.26	0.662
COPB	1.25	0.88 – 1.77	0.207	2.27	1.48 – 3.47	**<0.001**
Prior cardiac surgery	1.58	0.89 – 2.79	0.116	1.37	0.69 – 2.71	0.368
CB time (/10 min)	0.96	0.92 – 1.00	0.073	1.07	1.02 – 1.11	**0.002**
CABG	2.77	1.72 – 4.48	**<0.001**	2.19	1.19 – 4.02	**0.012**
Bimammary artery bypass	2.28	1.52 – 3.42	**<0.001**	1.34	0.78 – 2.32	0.292
Cardiac transplantation	1.03	0.23 – 4.60	0.968	8.69	3.27 – 23.07	**<0.001**
Post-bypass norepinephrine and dobutamine	2.42	1.68 – 3.50	**<0.001**	0.58	0.28 – 1.18	0.130
**VGA**	0.89	0.60 – 1.32	0.566	1.41	0.86 – 2.31	0.177

CABG, coronary artery bypass graft; CB, cardiopulmonary bypass; COPD, chronic obstructive pulmonary disease; ESRD, end-stage renal disease; SSI, surgical site infection; VGA, vancomycin gentamicin antibiotic prophylaxis.

Supplementary Table S6: Perioperative characteristics of patients according to the type of prophylaxis in the sensibility analysis restricted to patients with isolated CABG

	**Overall** N = 5,715 (100%)	**CA** N = 5,387 (94%)	**VGA** N = 328 (6%)	***p value***
**Demography**				
Male	4,831 (85)	4,598 (85)	233 (71)	<0.001
Age (years)	66 [58-73]	66 [58-73]	67 [60-74]	0.054
1st quartile < 56 years	1,096 (19)	1,040 (19)	56 (17)	
2nd quartile [56, 65] years	1,747 (31)	1,653 (31)	94 (29)	0.492
3rd quartile [66-74] years	1,673 (29)	1,571 (29)	102 (31)	
4th quartile >74 years	1,199 (21)	1,123 (21)	76 (23)	
BMI (kg/m^2)	26.9 [24.4-29.9]	26.9 [24.4-29.8]	27.4 [24.4-30.2]	0.292
Obesity	1,417 (25)	1,328 (25)	89 (27)	0.329
**Medical history**				
Smoking	2,537 (44)	2,379 (44)	158 (48)	0.156
Arterial hypertension	4,073 (71)	3,821 (71)	252 (77)	0.022
Diabetes mellitus	2,404 (42)	2,259 (42)	145 (44)	0.418
Insulin-dependent diabetes	817 (14)	755 (14)	62 (19)	0.014
Noninsulin-dependent diabetes	1,584 (28)	1,502 (28)	82 (25)	0.258
Dyslipidaemia	4,164 (73)	3,922 (73)	242 (74)	0.700
Chronic peripheral arterial insufficiency	1,117 (20)	1,046 (19)	71 (22)	0.323
Stroke	399 (7.0)	371 (6.9)	28 (8.5)	0.255
Cardiac insufficiency	283 (5.0)	262 (4.9)	21 (6.4)	0.212
Ischaemic heart disease	5,707 (100)	5,379 (100)	328 (100)	>0.999
ESRD	71 (1.2)	60 (1.1)	11 (3.4)	0.002
COPD	457 (8.0)	409 (7.6)	48 (15)	<0.001
Cirrhosis	29 (0.5)	27 (0.5)	2 (0.6)	0.682
**Preoperative data**				
B-blocker	4,699 (82)	4,436 (82)	263 (80)	0.320
ACE inhibitor	3,488 (61)	3,291 (61)	197 (60)	0.710
Statins	5,036 (88)	4,749 (88)	287 (88)	0.721
Antiplatelet agent	5,402 (95)	5,095 (95)	307 (94)	0.448
Aortic regurgitation	11 (0.2)	11 (0.2)	0 (0)	>0.999
Mitral regurgitation	14 (0.2)	14 (0.3)	0 (0)	>0.999
Aortic Stenosis	38 (0.7)	33 (0.6)	5 (1.5)	0.064
Mitral stenosis	3 (<0.1)	3 (<0.1)	0 (0)	>0.999
Surgical emergency	227 (4.0)	208 (3.9)	19 (5.8)	0.082
Acute infective endocarditis	1 (<0.1)	0 (0)	1 (0.3)	0.057
Prior cardiac surgery	49 (0.9)	44 (0.8)	5 (1.5)	0.202
Preoperative critical state				
MV	10 (0.2)	10 (0.2)	0 (0)	>0.999
Catecholamine	24 (0.4)	24 (0.4)	0 (0)	0.398

AKI	12 (0.2)	10 (0.2)	2 (0.6)	0.148
Haemoglobin (g/dl)	13.8 [12.6-14.8]	13.8 [12.7-14.8]	13.2 [11.9-14.3]	<0.001
Platelet count (G/l)	226 [190-271]	225 [190-270]	238 [196-293]	0.002
Prothombin ratio (%)	95 [87-100]	95 [87-100]	96 [87-100]	0.613
Creatinin (µg/L)	89 [76-106]	89 [76-105]	88 [76-110]	0.477
EuroSCORE II	1 [1-2]	1 [1-2]	2 [1-3]	<0.001
**Intraoperative data**	5,715 (100)	5,387 (100)	328 (100)	
ACC time	38 [32-47]	39 [32-47]	37 [31-44]	0.018
CBP time	47 [40-56]	47 [40-57]	46 [38-53]	0.027
Isolated CABG	5,715 (100)	5,387 (100)	328 (100)	
Bimammary artery bypass	5,206 (91)	4,907 (91)	299 (91)	0.966
CABG and valve surgery	0 (0)	0 (0)	0 (0)	
Isolated valvular surgery	0 (0)	0 (0)	0 (0)	
Thoracic aortic surgery	0 (0)	0 (0)	0 (0)	
Cardiac transplantation	0 (0)	0 (0)	0 (0)	
Post-bypass catecholamine	1,681 (29)	1,575 (29)	106 (32)	0.235
Post-bypass norepinephrine	812 (14)	784 (15)	28 (8.5)	0.002
Post-bypass norepinephrine and dobutamine	231 (4.0)	216 (4.0)	15 (4.6)	0.615
**Postoperative data**	5,715 (100)	5,387 (100)	328 (100)	
MV duration (hours)	6 [5-8]	6 [5-8]	6 [5-9]	0.052
Catecholamine duration (hours)	24 [7-44]	24 [7-43]	25 [10-48]	0.008
Blood loss at 24 H (ml)	640 [495-810]	640 [500-810]	560 [440-741]	<0.001
Total blood loss (ml)	810 [625-1,030]	810 [630-1,030]	750 [580-950]	0.004
Reintervention	464 (8.1)	425 (7.9)	39 (12)	0.010
Surgical site infection	372 (6.5)	340 (6.3)	32 (9.8)	0.014
Superficial SSI	295 (5.2)	270 (5.0)	25 (7.6)	0.038
Deep SSI	77 (1.3)	70 (1.3)	7 (2.1)	0.210
ICU LOS (days)	3 [2-4]	3 [2-4]	3 [2-5]	0.070
Total LOS (days)	8 [7-12]	8 [7-12]	9 [7-14]	0.033
D28 mortality	101 (1.8)	88 (1.6)	13 (4.0)	0.002
D90 mortality	154 (2.7)	134 (2.5)	20 (6.1)	<0.001

Supplementary Table S7: Multivariate analysis of risk factors for superficial and deep SSI in the sensitivity analysis restricted to patients undergoing isolated CABG

**Predictors**	**Superficial SSI**			**Deep SSI**		
	**Estimate**	**CI**	**p**	**Estimates**	**CI**	**p**
Male	0.32	0.25 – 0.42	**<0.001**	0.75	0.42 – 1.34	0.327
Age (years)						
1st quartile < 56 y	ref	ref	-	ref	ref	-
2nd quartile [56, 65] y	0.80	0.55 – 1.17	0.254	0.84	0.35 – 2.06	0.706
3rd quartile [66-74] y	0.84	0.57 – 1.22	0.357	1.81	0.81 – 4.03	0.147
4th quartile >74 y	1.13	0.76 – 1.68	0.534	2.53	1.12 – 5.73	**0.026**
Obesity	2.03	1.56 – 2.63	**<0.001**	2.52	1.55 – 4.10	**<0.001**
Insulin-dependent diabetes	4.27	3.09 – 5.91	**<0.001**	1.49	0.78 – 2.84	0.227
Noninsulin-dependent diabetes	2.47	1.81 – 3.38	**<0.001**	1.42	0.83 – 2.44	0.201
Dyslipidaemia	0.99	0.73 – 1.33	0.922	0.81	0.48 – 1.38	0.447
Chronic peripheral arterial insufficiency	1.84	1.39 – 2.43	**<0.001**	2.44	1.50 – 3.99	**<0.001**
Cardiac insufficiency	1.07	0.63 – 1.83	0.808	1.79	0.75 – 4.28	0.193
ESRD	2.26	1.10 – 4.63	**0.027**	0.75	0.10 – 5.72	0.784
COPB	1.13	0.74 – 1.73	0.563	2.29	1.26 – 4.16	**0.007**
Prior cardiac surgery	0.35	0.04 – 2.65	0.307	0.00	0.00 – Inf	0.980
CB time (/10 min)	0.96	0.90 – 1.03	0.280	1.09	0.98 – 1.21	0.109
Post-bypass norepinephrine and dobutamine	3.10	1.99 – 4.83	**<0.001**	0.44	0.10 – 1.90	0.273
**VGA**	1.22	0.77 – 1.93	0.395	1.40	0.62 – 3.16	0.417

CABG, coronary artery bypass graft; CB, cardiopulmonary bypass; COPD, chronic obstructive pulmonary disease; ESRD, end-stage renal disease; SSI, surgical site infection; VGA, vancomycin gentamicin antibiotic prophylaxis.

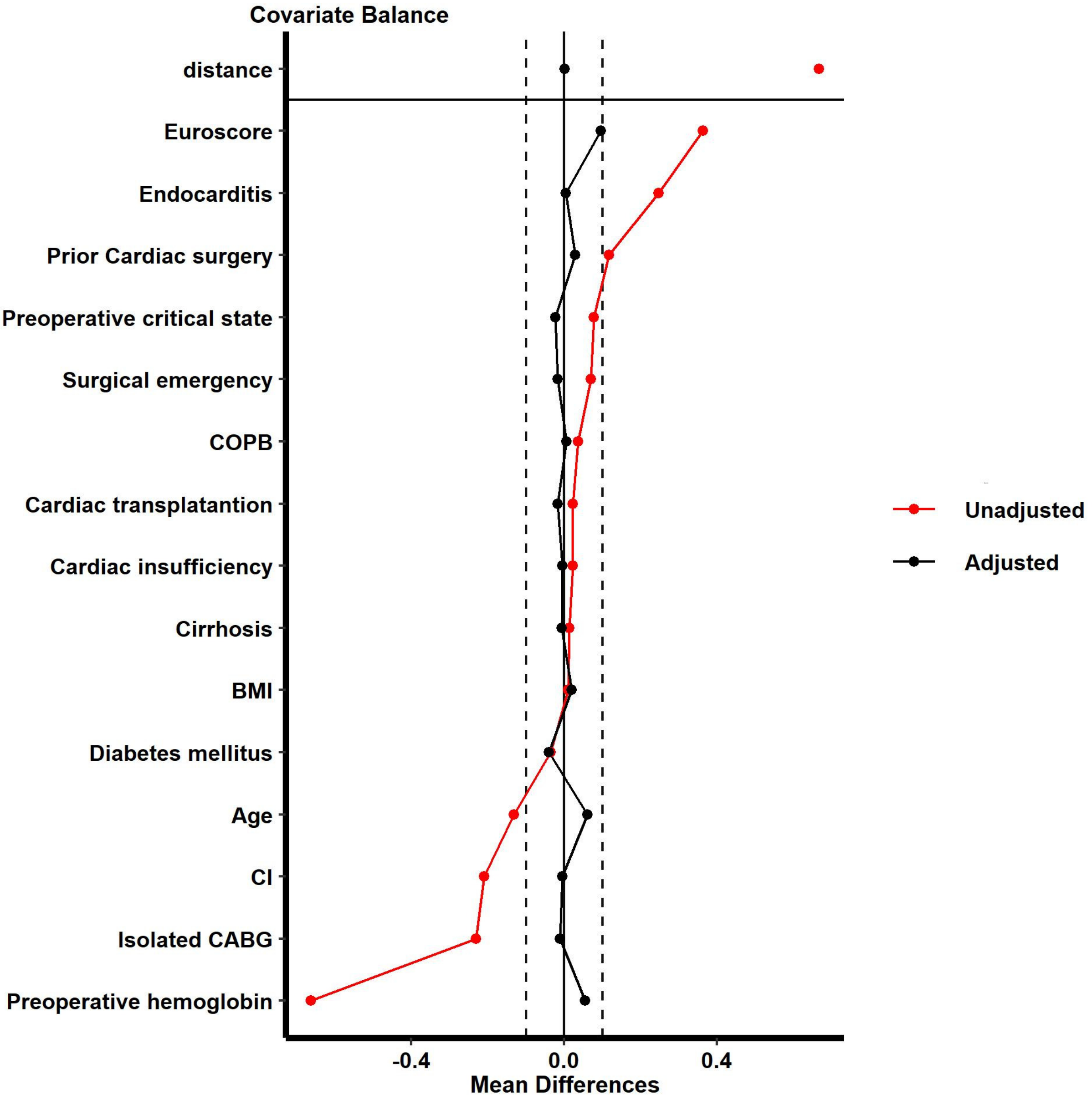

Supplementary Figure S1: Covariate balance in the propensity matched samples

Matching was performed using the Matching package[23], and covariate balance was assessed using cobalt[25]. Both packages were implemented in R (R Core Team, 2022).

BMI, body mass index, CABG, coronary artery bypass graft; CI, cardiac insufficiency; COPD, chronic obstructive pulmonary disease.

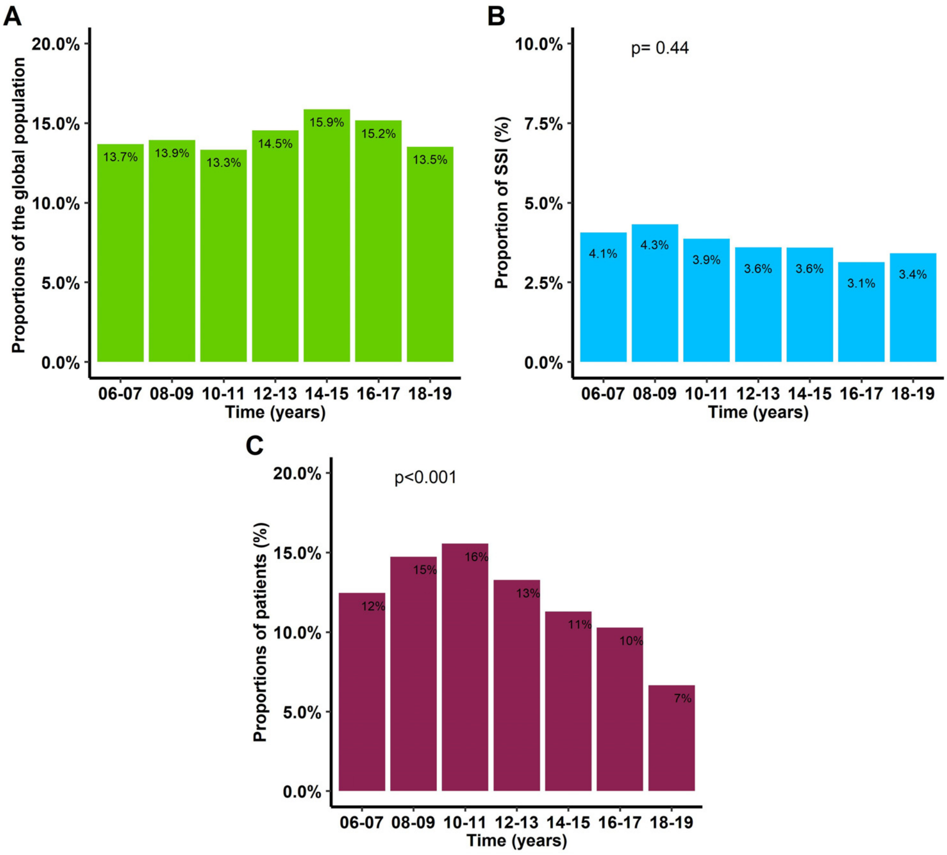

Supplementary Figure S2: Characteristics of the patients during the inclusion period

A, Proportion of patients included per two years during the study period.
B, Proportion of patients who presented a surgical site infection within the first 90 days after surgery per two years during the study period.
C, Proportion of patients treated with vancomycin/gentamicin every two years during the study period.

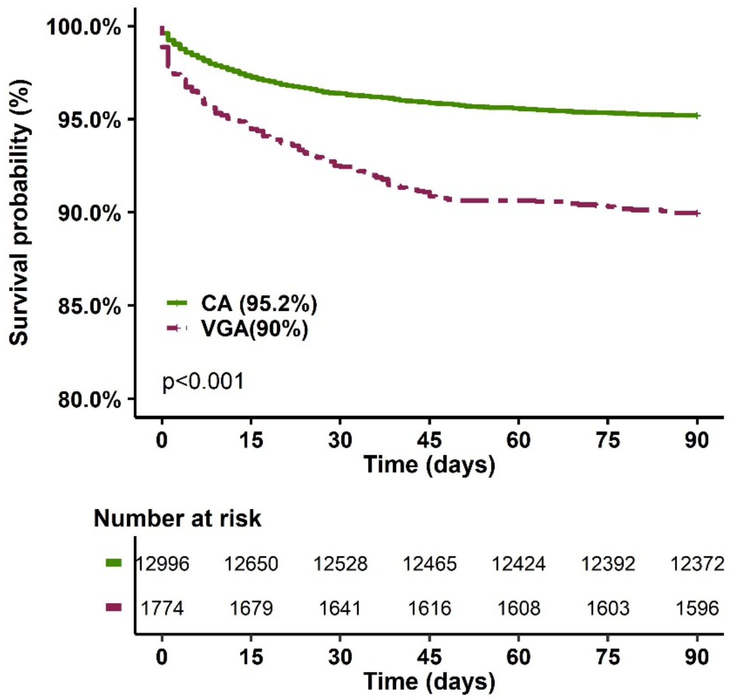

Supplementary Figure S3: 90-day mortality according to the type of prophylaxis

CA, cephalosporin antibiotic prophylaxis; VGA, vancomycin/gentamicin antibiotic prophylaxis.
